# Supplementary material for: Population-level comparisons of gene regulatory networks modeled on high-throughput single-cell transcriptomics data
Source: Nat Comput Sci. 2024 Mar 4;4(3):237–50. doi: 10.1038/s43588-024-00597-5 (PMC10965443; doi:10.1038/s43588-024-00597-5)
Supplement: Supplementary file 1 — Supplementary Figs. 1–4. [file 43588_2024_597_MOESM1_ESM.pdf]

nature computational science

Article
https://doi.org/10.1038/s43588-024-00597-5

Population-level comparisons of gene regulatory networks modeled on high-throughput single-cell transcriptomics data

In the format provided by the
authors and unedited

Supplementary Information

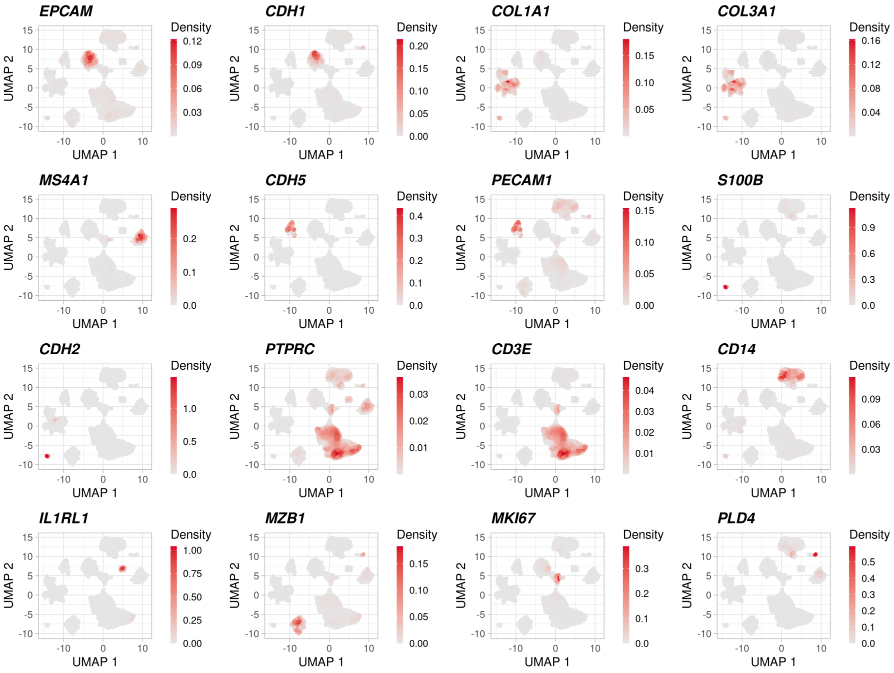

Supplementary Figure 1: Distribution of canonical markers used to annotate cell types in the colorectal cancer single-cell RNA-seq atlas through the Nebulosa package

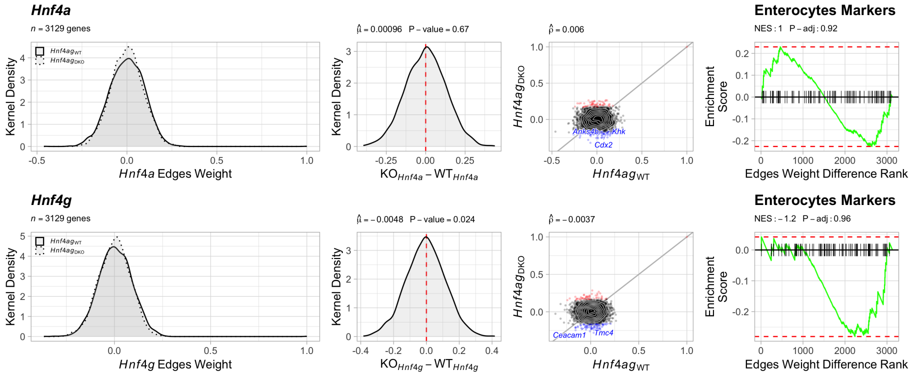

Supplementary Figure 2: Evaluation PPCOR's performance in transcriptome-wide scenarios using the same coarse-graining approach implemented in *SCORPION*. The first row presents results for the *Hnf4* α, and the second row for the *Hnf4* γ transcription factor. In the first panel, the distribution of edge weights is displayed, while the second panel shows the distribution of paired differences in edge weights between the networks constructed for the DKO and WT samples. The third panel illustrates the correlation between edge weights in both networks, and the fourth panel showcases the results of gene set enrichment analysis using the paired differences in edge weights between the DKO and WT sample networks.($\hat{\mu}$ and P-value were calculated using a one-sample t-test; NES and P-adjusted were computed using the GSEA test)

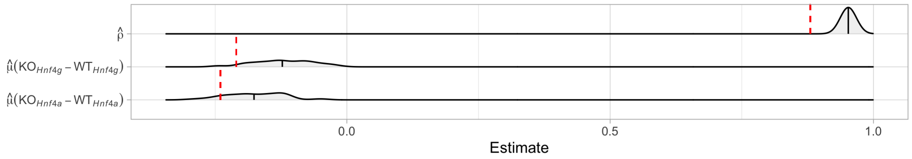

Supplementary Figure 3: Differences in results after employing random priors during the construction of single-cell gene regulatory networks with *SCORPION*. The presented density plots illustrate the distribution of the correlation ($\hat{\rho}$) between the edges' weight correlation for both *Hnf4*α and *Hnf4*γ transcription factors. Additionally, the distribution of the average pair differences between the edges' weight for *Hnf4*α: $\hat{\mu}\,(\mathrm{KO}_{Hnf4a} - \mathrm{WT}_{Hnf4a})$, and *Hnf4*$\gamma$: $\hat{\mu}\,(\mathrm{KO}_{Hnf4g} - \mathrm{WT}_{Hnf4g})$ transcription factors in networks constructed for the DKO and WT samples is shown. All distributions are based on 50 runs with random priors, with values obtained using correct priors highlighted in red.

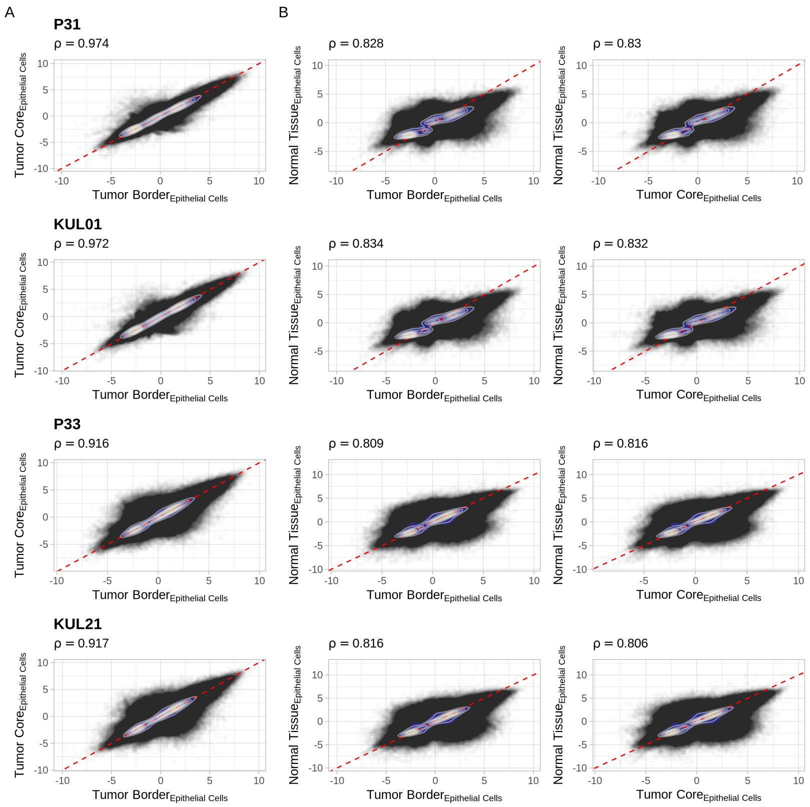

Supplementary Figure 4: Similarity (Spearman correlation coefficient) between gene regulatory networks generated from the same donor for different tumor regions. **(A)** Similarity between gene regulatory networks generated for border and core of the tumor cancer tissue.**(B)** Similarity between gene regulatory networks generated for the core of the tumor, border of the tumor and healthy adjacent tissue.
