# Supplementary material for: Population-level comparisons of gene regulatory networks modeled on high-throughput single-cell transcriptomics data
Source: Nat Comput Sci. 2024 Mar 4;4(3):237–50. doi: 10.1038/s43588-024-00597-5 (PMC10965443; doi:10.1038/s43588-024-00597-5)
Supplement: Supplementary file 2 — Reporting Summary [file 43588_2024_597_MOESM2_ESM.pdf]

nature portfolio

Corresponding author(s): Daniel Osorio; S. Stephen Yi; Marieke L. Kuijjer

Last updated by author(s): Jan 12, 2024

Reporting Summary

Nature Portfolio wishes to improve the reproducibility of the work that we publish. This form provides structure for consistency and transparency in reporting. For further information on Nature Portfolio policies, see our Editorial Policies and the Editorial Policy Checklist.

Statistics

For all statistical analyses, confirm that the following items are present in the figure legend, table legend, main text, or Methods section.

n/a	Confirmed	
☐	☒	The exact sample size (*n*) for each experimental group/condition, given as a discrete number and unit of measurement
☐	☒	A statement on whether measurements were taken from distinct samples or whether the same sample was measured repeatedly
☐	☒	The statistical test(s) used AND whether they are one- or two-sided<br>*Only common tests should be described solely by name; describe more complex techniques in the Methods section.*
☐	☒	A description of all covariates tested
☐	☒	A description of any assumptions or corrections, such as tests of normality and adjustment for multiple comparisons
☐	☒	A full description of the statistical parameters including central tendency (e.g. means) or other basic estimates (e.g. regression coefficient) AND variation (e.g. standard deviation) or associated estimates of uncertainty (e.g. confidence intervals)
☐	☒	For null hypothesis testing, the test statistic (e.g. *F*, *t*, *r*) with confidence intervals, effect sizes, degrees of freedom and *P* value noted<br>*Give P values as exact values whenever suitable.*
☒	☐	For Bayesian analysis, information on the choice of priors and Markov chain Monte Carlo settings
☒	☐	For hierarchical and complex designs, identification of the appropriate level for tests and full reporting of outcomes
☐	☒	Estimates of effect sizes (e.g. Cohen's *d*, Pearson's *r*), indicating how they were calculated

Our web collection on statistics for biologists contains articles on many of the points above.

Software and code

Policy information about availability of computer code

Data collection	Lee et al. accessible through GEO: GSE132465, and GSE144735. Qi et al. accessible through GSA: HRA000979, Qian et al. accessible through ArrayExpress: E-MTAB-8107, and Che et al. accessible through GEO: GSE178318. Gene expression quantification of the patient-derived xenografts generated for this study are available as Supplementary Table 8.
Data analysis	R 4.1.2<br>Seurat 4.4.0<br>Nebulosa 1.12.0<br>Rfast 2.1.0<br>TABIX 0.2.6<br>STAR 2.7.11

For manuscripts utilizing custom algorithms or software that are central to the research but not yet described in published literature, software must be made available to editors and reviewers. We strongly encourage code deposition in a community repository (e.g. GitHub). See the Nature Portfolio guidelines for submitting code & software for further information.

Data

Policy information about availability of data

All manuscripts must include a data availability statement. This statement should provide the following information, where applicable:

- Accession codes, unique identifiers, or web links for publicly available datasets
- A description of any restrictions on data availability
- For clinical datasets or third party data, please ensure that the statement adheres to our policy

The following datasets were used to construct the colorectal cancer single-cell RNA-seq atlas used in this study: Lee et al. accessible through GEO: GSE132465, and GSE144735. Qi et al. accessible through GSA: HRA000979, Qian et al. accessible through ArrayExpress: E-MTAB-8107, and Che et al. accessible through GEO: GSE178318. Gene expression quantification of the patient-derived xenografts generated for this study are available as Supplementary Table 8. All the generated networks are available as independent files at https://github.com/dosorio/SCORPION/Results/Networks. We also made available the unrefined networks for human (hg38) and mice (mm10) genes through the the following link: https://github.com/dosorio/SCORPION/Data.

Research involving human participants, their data, or biological material

Policy information about studies with human participants or human data. See also policy information about sex, gender (identity/presentation), and sexual orientation and race, ethnicity and racism.

Reporting on sex and gender	N/A. This study does not involve human research participants.
Reporting on race, ethnicity, or other socially relevant groupings	N/A
Population characteristics	N/A
Recruitment	N/A
Ethics oversight	N/A

Note that full information on the approval of the study protocol must also be provided in the manuscript.

Field-specific reporting

Please select the one below that is the best fit for your research. If you are not sure, read the appropriate sections before making your selection.

☒ Life sciences ☐ Behavioural & social sciences ☐ Ecological, evolutionary & environmental sciences

For a reference copy of the document with all sections, see nature.com/documents/nr-reporting-summary-flat.pdf

Life sciences study design

All studies must disclose on these points even when the disclosure is negative.

Sample size	Various experiments were conducted using public data, with reported sample sizes specified for each experiment. The sample sizes were determined based on the availability of public data.
Data exclusions	Multiple quality control filters were performed across experiments. Excluded samples are reported in the manuscript.
Replication	Two independent cohorts were used to replicate the findings from single-cell RNA-seq data
Randomization	The manuscript provides descriptions of the covariates used to categorize samples. These were primarily employed for conducting differential analysis between regions and sides of the disease.
Blinding	The research was conducted without blinding. Groups were essential for performing regression analysis and supervised comparisons between meaningful groups.

Reporting for specific materials, systems and methods

We require information from authors about some types of materials, experimental systems and methods used in many studies. Here, indicate whether each material, system or method listed is relevant to your study. If you are not sure if a list item applies to your research, read the appropriate section before selecting a response.

Materials & experimental systems

n/a	Involved in the study
☒	☐ Antibodies
☒	☐ Eukaryotic cell lines
☒	☐ Palaeontology and archaeology
☐	☒ Animals and other organisms
☒	☐ Clinical data
☒	☐ Dual use research of concern
☒	☐ Plants

Methods

n/a	Involved in the study
☒	☐ ChIP-seq
☒	☐ Flow cytometry
☒	☐ MRI-based neuroimaging

Animals and other research organisms

Policy information about studies involving animals; ARRIVE guidelines recommended for reporting animal research, and Sex and Gender in Research

Laboratory animals	Mus musculus; Nu/Nu mice, 5 to 6 weeks old (Envigo). Mice had unrestricted access to food and water and were group-housed in a controlled environment with temperature (21-22°C), humidity (40-51%), and light (12/12 light/dark cycle) within the vivariums.
Wild animals	N/A; This study did not involve wild animals.
Reporting on sex	N/A; This study did not perform comparisons based on sex.
Field-collected samples	N/A; This study did not involve field-collected samples.
Ethics oversight	The University of Texas at Austin and The University of Colorado Institutional Animal Care and Use Committee approved all animal procedures.

Note that full information on the approval of the study protocol must also be provided in the manuscript.

Plants

Seed stocks	*Report on the source of all seed stocks or other plant material used. If applicable, state the seed stock centre and catalogue number. If plant specimens were collected from the field, describe the collection location, date and sampling procedures.*
Novel plant genotypes	*Describe the methods by which all novel plant genotypes were produced. This includes those generated by transgenic approaches, gene editing, chemical/radiation-based mutagenesis and hybridization. For transgenic lines, describe the transformation method, the number of independent lines analyzed and the generation upon which experiments were performed. For gene-edited lines, describe the editor used, the endogenous sequence targeted for editing, the targeting guide RNA sequence (if applicable) and how the editor was applied.*
Authentication	*Describe any authentication procedures for each seed stock used or novel genotype generated. Describe any experiments used to assess the effect of a mutation and, where applicable, how potential secondary effects (e.g. second site T-DNA insertions, mosiacism, off-target gene editing) were examined.*
